# C-reactive protein: structure, function, regulation, and role in clinical diseases

**DOI:** 10.3389/fimmu.2024.1425168

**Published:** 2024-06-14

**Authors:** Hai-Hong Zhou, Yu-Long Tang, Tian-Hao Xu, Bin Cheng

**Affiliations:** ^1^ Centre for Translational Medicine, Gansu Provincial Academic Institute for Medical Research, Lanzhou, China; ^2^ Centre for Translational Medicine, Gansu Provincial Cancer Hospital, Lanzhou, China; ^3^ Centre for Translational Medicine, Sun Yat-sen University Cancer Center Gansu Hospital, Lanzhou, China; ^4^ Ministry of Education (MOE), Key Laboratory of Cell Activities and Stress Adaptations, School of Life Sciences, Lanzhou University, Lanzhou, China; ^5^ Key Laboratory of Preclinical Study for New Drugs of Gansu Province, School of Basic Medical Sciences, Research Unit of Peptide Science, Chinese Academy of Medical Sciences, Lanzhou University, Lanzhou, China

**Keywords:** C-reactive protein, acute phase, inflammation, structure, pentamer

## Abstract

C-reactive protein (CRP) is a plasma protein that is evolutionarily conserved, found in both vertebrates and many invertebrates. It is a member of the pentraxin superfamily, characterized by its pentameric structure and calcium-dependent binding to ligands like phosphocholine (PC). In humans and various other species, the plasma concentration of this protein is markedly elevated during inflammatory conditions, establishing it as a prototypical acute phase protein that plays a role in innate immune responses. This feature can also be used clinically to evaluate the severity of inflammation in the organism. Human CRP (huCRP) can exhibit contrasting biological functions due to conformational transitions, while CRP in various species retains conserved protective functions *in vivo*. The focus of this review will be on the structural traits of CRP, the regulation of its expression, activate complement, and its function in related diseases *in vivo*.

## 1 Introduction

Pentraxin (PTX) is a superfamily of multifunctional proteins that are highly conserved in evolution and is named because of its “pentagonal” structure under electron microscope observation. PTX originated early in evolution and is called the “living fossil”. Many forms of PTX in ancient horseshoe crabs about 250 to 300 million years ago (1, 2). As a superfamily protein, PTX has been conserved in phylogeny from arachnids to mammals. Its typical feature is the presence of a 200-amino acid pentraxin domain at its carboxyl end (3, 4), called the pentramerization protein domain. Also, all members of this family share a conservative sequence of 8 amino acids in the pentameric protein domain (His-x-Cys-x-Ser/Thr-Trp-x-Ser, where x can be any amino acid), called the pentameric protein tag, or the “signature sequence” of the pentameric protein (5).

The members of the PTX family identified so far mainly include C-reactive protein (CRP), serum amyloid protein P component (SAP), PTX3, PTX4, and neuronal PTX (NPTX), etc. (3, 5). Tillett and Francis first discovered CRP can react with C-polysaccharides in the pneumococcus cell wall in the presence of calcium ions (6). SAP is calcium-dependent recognition and binding of amyloid deposited in tissues. It is a highly conserved plasma glycoprotein in humans and all other species studied. Almost all amyloid deposits have been detected. SAP is present in both of them, which leads to a very high concentration of SAP in amyloid deposits, which can account for about 15% of the total mass (7). The amino acid sequence homology between SAP and CRP is as high as 51%. The structure observed under the electron microscope is highly similar, both showing a pentameric disc-like structure. SAP is considered to be directly related to CRP and originated from single gene duplication (8, 9). PTX3, as a secreted protein containing a pentameric protein domain, was first identified in fibroblasts, induced by IL-1 in endothelial cells, or produced by TNF stimulation (TSG14) in fibroblasts (10). The long-chain pentameric proteins with the same overall composition identified after PTX3 include guinea pig acrosome pentameric protein, neuronal pentameric protein (NPTX1, NPTX2), PTX4 and neuronal pentameric receptor (NPR). Among them, NPR is a transmembrane molecule with a transmembrane domain at its amino terminus (11, 12). CRP and SAP orthologs in different mammalian species have a high degree of sequence similarity. Still are significant differences in serum basal levels and changes during the acute phase reaction. CRP and SAP are the main acute phases in humans and mice respectively (13). PTX3 is highly conserved in humans and mice, while CRP and SAP, their sequence and expression regulation have evolutionary differences between mice and humans (13).

CRP is a typical short-chain PTX member, mainly synthesized in the liver to respond to inflammation. The most significant is the inflammatory stimulus signal mediated by the cytokine IL-6. Pentraxins recognize a wide range of exogenous pathogenic substances and mutated self-molecules, and behave as acute-phase proteins in a species-specific manner. The high degree of sequence homology and the appearance of the pentameric molecule that can be quickly identified indicate that all the different plasma proteins that bind to the classical pentameric ligands in a calcium-dependent manner belong to the same family member. Although the “long pentamer” contains moderately homologous sequence domains, it does not have the appearance of the pentamer. Also, calcium-dependent binding is necessary for the stability of secondary, tertiary and quaternary structures of most pentamers. It is also an essential element for the binding of pentamers to specific ligands, but “long pentamers” do not have such feature.

CRP is an acute-phase protein primarily synthesized in liver hepatocytes and plays a critical role in inflammation response (14–17). When the body is invaded by pathogens such as bacteria, fungi, parasites or subjected to inflammatory stimuli, as well as after tissue damage caused by trauma and progressive cancer, the plasma concentration of CRP can increase rapidly within 6–8 hours and reach the peak in about 48 hours, rising 1000-fold from the basal level ~ 0.5 μg/ml (18). It will return to baseline once the inflammation subsides (19). The plasma levels of CRP exhibit a positive correlation with the severity of inflammation, making it a commonly employed nonspecific biomarker in clinical applications (20–22).

Furthermore, CRP acting as an innate pattern recognition receptor (23), can precipitate the somatic C-polysaccharide of *Streptococcus pneumoniae* on microorganisms in the presence of calcium, then triggers the innate immunity through the classical complement pathway by interacting with the global head region of C1q or other cell surface glycoproteins such as FcγR receptors (16, 24, 25). The structural analysis of the CRP binding phosphocholine (PC) ligand and C1 complex using Cryo-Electron Tomography (Cryo-ET) revealed a novel mechanism. Contrary to previous understanding, it was observed that CRP does not form an Fc-mediated hexamer antibody platform to bind and activate the C1 complex. Instead, CRP forms a rectangular platform assembled by tetrameric CRP to effectively bind and activate complement (25).

## 2 CRP structure-related features

**Figure 1:**
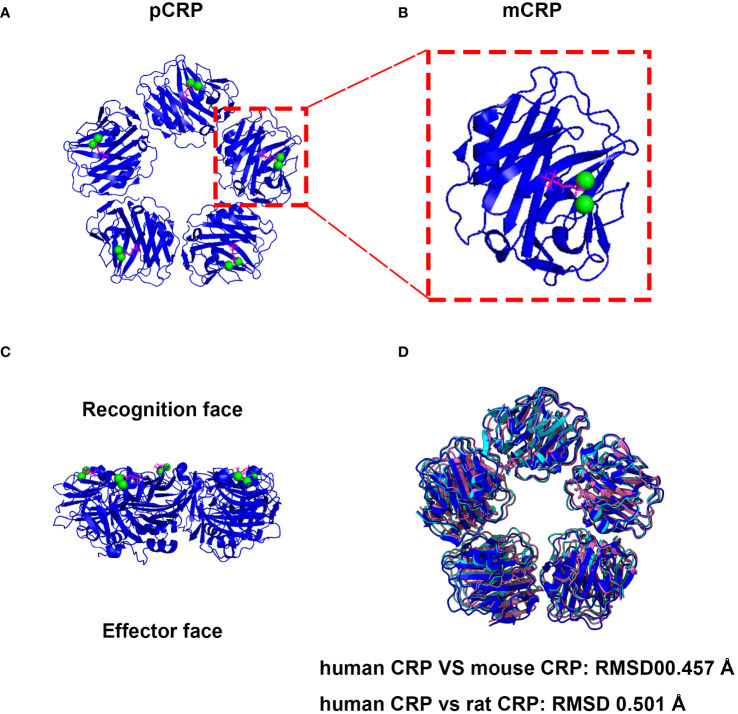
The native structure and associated activities of CRP are evolutionarily conserved. **(A)** Cryo-EM structure of CRP complexes with PC. The CRP-PC complex was achieved from Protein Data Bank (PDB entry 7PKE) and the ribbon diagram of the CRP Cryo-EM structure of CRP-PC-Ca^2+^ was generated by ChimeraX software (27). **(B)** View of the ligand binding face of mCRP. Each protomer contains a binding site, which is shown occupied by 2 calcium (green) and 1 PC molecule (magenta). **(C)** Side view of the pentameric form of CRP. Which mediates PC ligand-binding named recognition face, and the other side possesses a single α-helix, which binds to ligands such as C1q, therefore named effector face. **(D)** The pentameric form structures of mouse and rat CRP were generated with AlphaFold 3 using five subunits (28).

Human CRP (huCRP) has a molecular weight of approximate 115 kDa and is characterized by a “jelly-like lectin fold”. Generally, CRP is composed of five identical subunits which assemble into a cyclic pentamer around a central pore (26). One side within each promoter contains a calcium-binding pocket, accommodating two calcium irons to stable its structure, which further mediates PC ligand-binding named recognition face (**Figure 1**). The other side possesses a single α-helix, which binds to ligands such as C1q, therefore named effector face (**Figure 1**). HuCRP is non-glycosylated and has no inter-subunit covalent bond, while a sole disulfide bond within each subunit formed between Cys36 and Cys97. The formation of disulfide bonds in hCRP protomers should occur in the early spontaneous folding stage driven by the conformational folding of six β strands, which plays a vital role in the subsequent conformational reconstruction of the whole subunit and the pentamer assembly (29).

### 2.1 CRP-ligand affinity

The physiological function of CRP is related to calcium-dependent ligand affinity (30, 31). Many studies have used calcium-dependent PC affinity to successfully separate CRP from blood, such as horseshoe crab (32), mouse (33), dog (34), cat (35), cow (36), since the calcium-dependent PC affinity is conserved in the species and as a basis for testing the function of multi-species CRP in experiments. The CRP of human, mouse and rat were expressed in different systems and purified based on the calcium-dependent binding properties to PC (37–40). The binding ability of pentamer CRP to pattern recognition ligand PC was no significant difference among different species (38). In addition, CRP not only can bind to various pathogens, including fungi, yeasts, and bacteria, but also has calcium-dependent binding properties to chromatin, histones, and small ribonucleoprotein U1, and glycans (41). In the absence of calcium, CRP can bind to polycations such as poly-L-lysine, poly-L-arginine, and myelin essential protein (42).

### 2.2 Structural analysis of CRP

Shrive team first obtained the X-ray crystal structure of huCRP in a calcium-binding state (PDB ID: 1GNH) (43). Thompson group further resolved huCRP in PC-binding state in the presence of calcium (PDB ID: 1B09) (44). It reveals that Phe-66 and Glu-81 are two key residues that mediate the binding of PC to CRP. The distance between the point and the calcium ion is only 0.4 nm (45). Among them, Phe-66 provides hydrophobic interaction with the methyl group of PC, while Glu-81 is located at the other end of the pocket and interacts with the positively charged choline nitrogen (46). Ramadan et al. solved huCRP in a calcium-depleted state (PDB ID: 1LJ7) (26), revealing a decamer with two independent pentamers stacked near parallel in a face A-to-face A form. In 2014, Guillon et al. obtained two crystal structures of huCRP, assembled in a staggered decameric form (47). One of them is stabilized by two zinc ions trapped within a cleft on the effector’s face, also in the presence of HIV-1 Tat protein (PDB ID: 3PVN).

With the rapid development of Cryo-Electron Microscopy (Cryo-EM) and artificial intelligence (AI), it is more convenient and fast to analyze and predict the three-dimensional conformation of protein space. Noone et al. used Cryo-EM to analyze the structure of CRP at pH 7.5 or pH 5 and in the presence or absence of the ligand PC, and found that the previous structure obtained from crystallography was an imperfect pentagon with variable angles between each subunit (48). The pentamer CRP obtained by Cryo-EM was found to have C5 symmetry, and the subunit was formed into an isometric regular pentagon (48). Subsequently, they applied the Cryo-ET resolved the structure of CRP binding to PC after binding to C1 complex. Revealed new interfaces, interpreting previously contradictory biochemical data, and clarifying the possible mechanism by which CRP regulates the complement system (25).

### 2.3 Conformational forms of CRP

Generally, huCRP primarily exists in two conformational forms. One is the native homo-pentameric form, namely pentameric CRP (pCRP) or native CRP (nCRP), the other is the dissociated form, namely monomeric or modified CRP (mCRP) (49–54). Pentameric CRP can undergo continuous structural changes to form mCRP under various conditions, including low pH, absence of calcium, increased temperature, urea chelation, or binding to activated platelets, apoptotic/necrotic cells, and microparticles (55). It is commonly accepted that pCRP responds to tissue damage or infection by rapidly increasing its blood concentration. Accumulating evidence suggests that pCRP dissociates into mCRP and that most of the proinflammatory effects of CRP are expressed only after dissociation of its native pentamer assembly into mCRP.

It has been shown that CRP exhibits a rapid pentamer-decamer equilibrium at 2 mM Ca^2+^, with the proportion of decamers decreasing as NaCl concentration increases (56). Both pH and the presence of PC appear to affect the ratio of CRP pentamers to decamers, as well as particle orientation within glass ice, with a pH 5 buffer producing more decamers than a pH 7.5 buffer (48). Based on site-directed mutagenesis studies of amino acids at specific sites, Agrawal and Volanakis proposed that the interaction between CRP and C1q is dependent on conformational changes in the CRP pentamer (57). Through the ligand binding ability under simulated acidic conditions, it was found that the ligand binding was significantly increased at pH 5, and the binding to immobilized C1q and immunoglobulin was also increased. That is, subtle changes in charged residues on CRP and immune ligands can significantly increase their association with inflammatory stimulus response, potentially activating the downstream immune effector function of CRP (48).

Monomeric CRP has been considered as a major contributor in local inflammation (54, 55, 58). Not only it can induce pro-inflammatory effects on cells such as monocytes and endothelial cells (59), and trigger the secretion of interleukin-8 (IL-8) by human neutrophils (60), but also promote inflammation of the human coronary artery endothelial cells phenotype through a p38 mitogen-activated protein kinase-dependent mechanism (61) and enhance neutrophil localization and activation at inflamed or injured vascular sites (62, 63). In addition, mCRP can promote neutrophil-endothelial cell adhesion and delay apoptosis of human neutrophils (64). With accumulating in human atherosclerotic lesions, mCRP can induce monocyte chemotaxis, promote neutrophil survival, which may amplify the inflammatory response (52, 65).

## 3 Regulation of CRP expression

The huCRP gene is located in the 1q23–24 region of the chromosome. It encodes 224 amino acids, of which 18 amino acids at the N-terminus are the signal peptide sequence. The whole gene contains only one intron, which separates the region into encoding the signal peptide and the mature protein (66). CRP responds to IL-6 and IL-1β combined stimulation that can produce an acute phase typically. The promoter region contains two acute-phase response elements, including a binding site for the liver-specific transcription factor hepatocyte nuclear factor (HNF1) and two C/EBPβ (CCAAT/enhancer-binding protein β) binding sites for interleukin-6 (IL-6) induced transcription (67, 68).

The expression of CRP is tissue-specific and is mainly produced by hepatocytes. However, it can also be synthesized in monocytes, neuronal cells, lymphocytes, and atherosclerotic plaques, although CRP produced in those cells or tissues does not contribute to the serum level (69). Its serum concentration is significantly increased in inflammatory diseases such as cardiovascular complications (70). CRP plasma base level can be affected by many factors, such as chronic infectious diseases caused by pathogen invasion or smoking, body mass index (BMI), coffee intake, oral contraceptives, and genetic factors, etc. (71).

The synthesis of CRP in hepatocytes is mainly regulated at the transcription level by the stimulator cytokine IL-6 and interleukin-1β (IL-1β) (67, 72). Transcription factors, such as STAT3, Rel p50, c-Rel, and C/EBPβ/δ are mainly involved in the regulation of CRP expression. STAT3 and Rel bind adjacent to the CRP promoter in the non-coding region, allowing closer binding of C/EBP, an important signaling molecule, to the nucleic acid and promoting its efficiency in inducing CRP expression (73). The NF-κB binding site is located at -69 in the proximal CRP promoter, overlapping with the transcription factors OCT-1/HNF-1/HNF-3 binding sites. IL-1β induces CRP transcription through the proximal CRP promoter by activating NF-κB-p50/p65 in synergy with IL-6-activated C/EBPβ (74). In the absence of C/EBPβ, a complex containing C/EBPδ and RBP-Jκ is formed at the C/EBP-p50 site. The synergistic effect of IL-6 and IL-1β in the induction of CRP gene expression is partially mediated through the NF-κB locus (74).

OCT-1 can inhibit CRP transcription induced by IL-6 and IL-1β through the proximal CRP promoter (75). In addition, post-transcriptional level regulation also plays a particular role in CRP expression, especially in the acute phase when CRP is secreted in large amounts. Under normal physiological conditions, CRP expression is at a very low level. Most CRP is bound to carboxylesterase and stored in the endoplasmic reticulum (ER) (76). In the acute phase, the conformation of carboxylesterase changes, which significantly reducing the binding capability with CRP, thus CRP is released from ER (77).

The expression pattern of CRP is causally determined by the promoter methylation status tuned by DNMT3A and TET2 (21). The CpG deficient promoter motif of CRP is located at the binding sites of STAT3, C/EBP-B, and NF-κB. These motifs are highly methylated in the resting state but undergo STAT3 and NF-kB-dependent demethylation in response to cytokine stimulation, leading to a significant increase in C/EBP-B, thereby promoting CRP expression (21). Further analysis showed that reversible methylation could also regulate highly induced gene expression with CpG promoters represented by APRs. Thus, these promoters lacking CpG may evolve TF binding sites containing CpG, utilizing dynamic methylation to achieve a rapid and reversible response (21, 78).

Natural conformational CRP is the dominant conformation secreted by most cell types, but some cells also directly release mCRP. When mCRP is injected into circulation can be rapidly redistributed into tissues through a lipid raft mediated mechanism. However, the transport of mCRP locally produced in the inflammatory microenvironment into the circulation remains unclear (79, 80).

## 4 Functional differences of CRP in humans and experimental animals

Currently, research on the impact of human CRP has predominantly involved introducing human CRP into mouse and rat models either through transgenic methods or direct injection (72, 81, 82). It is recognized that CRP behaves differently in mice and rats compared to humans, particularly in its role as an acute phase protein and its interaction with the complement system (83). This variation has led to the consideration of these animal models as naturally deficient in CRP expression or function, thus rendering their endogenous CRP activity negligible. However, whether CRP can activate auto-complement in rats is still controversial, and there are concerns that serum CRP levels in mice may be inaccurately measured (84). Human CRP recognizes microorganisms and apoptotic cells by binding to PC and promotes phagocytosis of phosphorylated substances by activating the classical pathway of complement (CP) (9). In addition, CRP inhibits complement hyperactivation by binding to complement factor H (CFH), the major inhibitor of the alternative pathway (AP) (85). While human CRP is known to adopt an activated conformation and interact with C1q and CFH to activate or inhibit complement, respectively (86), doubts have been raised about the conservation of these interactions in rodents (86, 87). Our recent findings show that mouse, rat, and human CRPs all exhibit complement activating capacity, binding to their own C1q and activating their respective classical complement pathways alone (38, 86).

Moreover, endogenous CRP knockout in mice and rats, and human CRP complementation (intravenous injection and gene knock-in) showed consistent significant phenotypes in acute liver injury models that contribute significantly to complement activation. CRP has a protective effect on acute liver injury and is able to delay death caused by sepsis, indicating that CRP plays an important protective function and is conserved among species (38, 86). It is further pointed out that endogenous CRP in mouse and rat animal models should not be ignored when studying the function of human CRP. This suggests that the research paradigm and experimental design of relevant animal models need to be reconsidered and optimized.

## 5 CRP and clinical diseases

Until now, no human CRP gene defects have been reported, nor even any sequence polymorphisms in the protein itself. Although their actual function in humans is unknown, gene deletion studies in mice suggest that CRP contribute to innate immunity. CRP is the quintessential human acute-phase protein that is used in clinical practice around the world to monitor disease activity. A growing number of clinical trials have shown that CRP is related to many diseases. In cardiovascular research, CRP has been used to diagnose cardiovascular disease (CVD) and as a marker to indicate disease status and incidence (88). Besides, CRP is associated with atherosclerotic vascular disease (ASVD), systemic lupus erythematosus (SLE), cancer, and other diseases (14, 89–91). Its influence on these diseases occurrence, development, and prognosis should not be underestimated (92, 93). CRP is also related to the epidemic virus COVID-19 and is an independent predictor of mortality from COVID-19 infection (94–97).

### 5.1 CRP and innate immunity

CRP participates in the innate immune response as a pattern recognition receptor, and each of five homologous globular subunits has a calcium-regulated binding pocket for ligands expressing the PC moiety. Upon binding to ligands on damaged and apoptotic cells, it undergoes a conformational change that allows binding and activation of C1 and may lead to dissociation of monomeric CRP (81). Although CRP recognition of PC or related molecules on microorganisms plays an important role in our defense, a more important role may be the binding of CRP to PC in damaged membranes. PC are not normally exposed to the cell surface but are exposed due to damage by complement or certain phospholipases (98). CRP binds to apoptotic and necrotic cells, and defective clearance of apoptotic cells is associated with autoimmune diseases (81). The colocalization of CRP with fixed complement in areas of tissue injury suggests that CRP may play a role in clearing cellular debris from tissue. Mark et al. showed that CRP had inherent pro-inflammatory effects, either on human peripheral blood mononuclear cells *in vitro* or when administered parenterally to mice or healthy human volunteers *in vivo* (99, 100). Genetic and epigenetic studies of gene targeting mice and humans have shown that CRP plays a crucial non-redundant function in innate immunity, inflammation, and tissue remodeling. The innate immune response is activated when the conserved structure on the pathogen surface, the pathogen-associated molecular pattern (PAMP), is recognized by CRP, a pattern recognition molecule (PRM) (101).

For CRP, the mouse is not an ideal model as its CRP levels do not respond to inflammatory stimuli (102). Several studies have been performed to assess the role of huCRP in transgenic mice overexpressing huCRP. Those studies found that huCRP facilitates the survival of mice infected with *S. pneumoniae* (103, 104). This effect is mediated mainly by the strong response of CRP to the PC present in the cell walls of these bacteria (105). These data indicate that CRPtg mice infected with *S. pneumoniae* are resistant to infection, showing longer survival time and lower mortality than non-transgenic littermates (wild type) (106). Similar studies have shown that CRP administration can prevent the invasion and infection of *Haemophilus influenzae* (107). CRPtg animals can resist infection by the Gram-negative pathogen *Salmonella* even without CRP binding (108).

### 5.2 CRP and CVD

CVD is one of the major diseases threatening human health today, mainly caused by atherosclerosis (AS). Cardiovascular risk factors such as obesity, hypertension, diabetes, smoking, and dyslipidemia can lead to intravascular inflammatory response and endothelial cell activation and dysfunction, the earliest event in the development of AS. The most widely studied inflammatory marker related to CVD is CRP. Elevated CRP levels are directly proportional to CVD risk and are an independent risk factor for cardiac death. By measured serum CRP and creatine kinase levels in patients with CVD but without cardiogenic chest pain. The results showed that all individuals with acute myocardial infarction showed elevated CRP secretion levels, and there was a significant correlation between peak CRP concentration and creatine kinase values (109, 110). Mild, 2 - to 5-fold increases in baseline plasma CRP levels in asymptomatic individuals are associated with an increased risk of cardiovascular events such as stroke and myocardial infarction (111, 112). And the use of mildly elevated CRP levels to guide primary prevention has led to a significant reduction in major cardiovascular events in apparently healthy persons (113). Although the exact role of CRP in atherosclerosis and its complications remains unclear, there is now increasing evidence that it may be a direct causative factor (9, 114). The crucial role of CRP in the prevention, treatment, and prognosis of CVD has been agreed upon and is even used as the “gold standard” for CVD risk assessment (115, 116).

### 5.3 CRP and cancer

CRP is a human acute-phase protein, and its plasma level is associated with cancer risk (117, 118). In clinical practice, the CRP test is widely used to monitor disease severity, the clinical course of the disease, and treatment responses. Recent studies have strongly suggested that CRP acts as a pivotal contributor to the development and progression of tumors. Increased CRP level is reported in colorectal, lung, and gastric cancer cases (50). The host interacts with tumor factors, and these interactions can accelerate tumor progression or regression. Lymphocyte-to-CRP ratio (LCR) has been used as a post-surgical prognostic biomarker in gastric and colorectal cancer (119). Studies have shown that CRP reduction is an early predictor of post-operative complications of gastric cancer and a reliable discharge indicator after gastric cancer (120, 121). Elevated preoperative CRP predicts increased post-operative morbidity in a patient with colorectal neoplasia (122). Anastomotic leakage is associated with higher CRP levels each post-operative day than no anastomotic leakage after colorectal surgery. The cut-off CRP values can be used to predict anastomotic leakage to expedite investigation and treatment (123).

Inflammation in the tumor microenvironment plays a vital role in cancer invasiveness, progression, and metastasis. Preoperative CRP levels help to diagnose differentiated thyroid carcinoma (50, 124). CRP has been reported to be consistently increased in the circulation of patients with body wasting associated with chronic diseases. In addition, CRP as an enhancer of *in vitro* IgG-mediated erythrocyte and tumor cell destruction (125). Simultaneously, CRP is a highly sensitive marker of inflammation to be considered in diagnosing cancer cachexia (126). Moreover, CRP levels are very responsive to lifestyle and several pathophysiological conditions. Thus, identifying cut-off values for CRP values is needed and should consider the heterogeneity of cancer patients’ clinical profiles.

### 5.4 CRP and SLE

CRP is associated with the binding and regulation of SLE associated nuclear antigens such as chromatin, histones, small nuclear ribonucleoprotein U1, and apoptotic cells (127). Several studies have shown delayed disease progression and increased survival in NZB/NZW mice carrying huCRP transgene, a protective effect associated with CRP’s ability to limit kidney damage by preventing immune complex deposition (128). SLE patient sera containing high levels of pCRP had an inhibitory effect on type I IFN induction compared with patient sera containing low levels of pCRP (58).

The CRP subunit is similar to pCRP and mCRP, but its monomeric structure exposes protein regions hidden in pentameric forms, including hidden protein neoepitopes (129, 130). Autoantibodies against these neoepitopes on mCRP have been demonstrated in SLE patients. The inflammatory environment can induce dissociation of pCRP into mCRP, which may be a way to limit PCRP-induced inflammation. In addition, mCRP has been shown to promote the removal of immune complexes (ICs) (131). Both mCRP and pCRP induced low levels of TNF and IL-1β without the need for immune complexes (ICs) (58). The genetic association between non-coding polymorphisms in CRP and human susceptibility to SLE has not been established. Autoantibodies against CRP are often present in patients with lupus nephritis (LN). Amino acid residues 35–47 constitutes the major epitope recognized by anti-CRP autoantibodies in patients with LN. Anti-a.a.35–47 autoantibodies are closely associated with renal prognosis, suggesting a pivotal role for mCRP in LN (132).

### 5.5 CRP and virus infection

CRP is one of the most frequently tested molecules in clinical medicine. In daily practice, it is used for nonspecific initial diagnosis of viral or bacterial infections and also for monitoring the course of such infections under drug therapy (87). Severe coronavirus disease 2019 (COVID-19) is a public health emergency due to its high infectiousness (133) and high morbidity and mortality rate in critically ill patients. COVID-19 has been associated with inflammation in its induced neurological, cardiovascular, and other end-organ. It is indispensable to explore biomarkers to assess the extent of lung disease and the severity of COVID-19 (134–136). Plasma levels of CRP can be used for the early diagnosis of pneumonia (137), independent of patient age, gender, and physical condition, and correlate with inflammation levels (138), higher in patients with severe pneumonia. Thus, it is an important indicator for diagnosing and evaluating severe pulmonary infectious diseases (139). Some studies have shown that CRP levels in early COVID-19 are positively correlated with lung lesions, and CRP levels can reflect disease severity and should be used as a critical indicator for disease monitoring (140). Based on this, several studies have evaluated CRP levels in COVID-19 patients and their risk of death. Importantly, CRP plasma levels were generally significantly lower in viral infections than in bacterial infections (141). This is particularly important when studying diseases caused by COVID-19. Before COVID - 19 pandemic, as much as 90% of CRP were significantly elevated due to infectious etiology, is one of the most common bacterial pathogens (142). CRP levels are alarmingly high in COVID-19 patients with poor prognosis, despite their viral illness. Plasma concentrations as high as 400 mg/l are usually seen only in severe bacterial infections or sepsis, often in harmful COVID-19 pneumonia without superinfection (143).

Study shows that the magnitude of CRP upregulation exhibited by patients with the severe acute respiratory syndrome (SARS) was associated with their respiratory dysfunction and death (140, 144). Besides, CRP levels were also significantly elevated in patients with the Middle East respiratory syndrome (MERS) and H1N1 influenza (94, 145–147). Based on this, several studies have evaluated CRP levels in COVID-19 patients and their risk of death (148). One of the results showed that CRP levels were significantly higher in deceased patients than surviving patients (95).

Although elevated CRP blood level is associated with death caused by COVID-19, results are inconsistent across populations (149, 150). CRP is closely related to the grade of disease severity caused by the COVID-19, oxygenation rates, radiological evidence of ARDS, and level of respiratory support (134). CRP blood level rise of COVID-19 patients in their first 7 days of hospitalization can predict disease progression and serve as a basis for the need to transfer patients to the ICU at an early stage. The median value of CRP correlates with the severity of COVID-19 and is an independent predictor of mortality caused by COVID-19 (95). However, whether the determined CRP threshold can be used for early risk stratification of patients and to guide intensive management of respiratory support and corticosteroid immunosuppression still needs to be determined in future prospective studies.

## 6 Conclusions and prospects

At present, the animal models used to study the function of human CRP mainly rely on mice and rats, whose serum CRP baseline levels and inflammatory stress levels are very different from those of humans. The baseline CRP concentrations in mice was trace, ~7.5 μg/ml, which rise only twofold in the acute phase response (151), and have been reported to be a minor acute phase reactant in response to inflammatory stimuli (152). In healthy and pathogen-free rats, CRP ranges from 300–600 µg/ml (153). After injection of casein or croton oil, rising 3- to 4-fold in the acute phase response, it rose only up to about 900 µg/ml, indicating that it is a poor marker of acute inflammation. In humans, the median baseline CRP concentration is 0.8 µg/ml, and can rise to > 500 µg/ml at the peak of the acute phase response.

Nevertheless, the sequence homology of CRP among different species is relatively high, its structural characteristic may vary as indicated by its different *in vivo* function and the available structure information. Minor alteration in the pentameric assembly and subunit conformation of mouse, rat, and human CRP by electron microscopy visualization, single-particle analysis, and homology modeling (38). These findings suggest that various CRP may have different functions in different species, and therefore a possible functional role of CRP in humans cannot be reliably inferred from experimental animal studies.

Recent findings indicate that pCRP can be separated by platelets in areas of inflammation, leading to the deposition of mCRP. mCRP can exert local pro-inflammatory effects through a variety of mechanisms, which have been studied before. Therefore, the local formation of mCRP in the inflammatory area may represent the “activation signal” of other inflammatory cells, and mCRP may help stimulate the local inflammatory process.

However, the dissociation process of pCRP is still not fully understood, and more experimental models need to be designed to help develop and test potential therapeutic mCRP blockers or pCRP-dissociation blockers. Future research needs to further solve the problem of mCRP receptors and signal transduction. Conclusive animal data on the role of mCRP and the dissociation process of pCRP are still lacking, and it is necessary to establish animal models of acute and chronic inflammation (including AS) suitable for CRP research. This will enable the confirmation of existing *in vitro* data and therapeutic strategies for the dissociation process of mCRP or pCRP.

Circulating CRP concentration correlates with the severity, degree, and progression of many different pathologies and the prognostic significance of these correlations, which is consistent with CRP as a marker of disease and contributes to the pathogenesis. Understanding the structure and function of CRP, including its 3D structure and its complex with ligand can establish a good platform for drug design. In view of the problem that endogenous CRP in animal models cannot be ignored, the design of animal models using mice and rats as animal models to study the function of human CRP should be reviewed as soon as possible in the field, and the experimental design should be optimized to further clarify the role and function of CRP in diseases.

## Author contributions

H-HZ: Conceptualization, Funding acquisition, Investigation, Writing – original draft. Y-LT: Data curation, Investigation, Visualization, Writing – original draft. T-HX: Data curation, Investigation, Visualization, Writing – review & editing. BC: Conceptualization, Data curation, Investigation, Validation, Visualization, Writing – original draft, Writing – review & editing.
